# Ti nanorod arrays with a medium density significantly promote osteogenesis and osteointegration

**DOI:** 10.1038/srep19047

**Published:** 2016-01-08

**Authors:** Chengyun Ning, Shuangying Wang, Ye Zhu, Meiling Zhong, Xi Lin, Yu Zhang, Guoxin Tan, Mei Li, Zhaoyi Yin, Peng Yu, Xiaolan Wang, Ying Li, Tianrui He, Wei Chen, Yingjun Wang, Chuanbin Mao

**Affiliations:** 1 School of Materials Science and Engineering, South China University of Technology, Guangzhou 510641, China; 2 Department of Chemistry & Biochemistry, Stephenson Life Sciences Research Center, University of Oklahoma, 101 Stephenson Parkway, Norman, Oklahoma 73019-5300,United States; 3 General Hospital of Guangzhou Military Command of PLA, Guangzhou 510010, China; 4 Institute of Chemical Engineering and Light Industry, Guangdong University of Technology, Guangzhou 510006, China; 5 School of Materials Science and Technology, Kunming University of Science and Technology, Kunming 650093, China; 6 School of Materials Science and Engineering, Zhejiang University, Hangzhou, Zhejiang 310027, China

## Abstract

Ti implants are good candidates in bone repair. However, how to promote bone formation on their surface and their consequent perfect integration with the surrounding tissue is still a challenge. To overcome such challenge, we propose to form Ti nanorods on their surface to promote the new bone formation around the implants. Here Ti nanorod arrays (TNrs) with different densities were produced on pure Ti surfaces using an anodizing method. The influence of TNr density on the protein adsorption as well as on the adhesion, proliferation, and osteogenic differentiation of MC3T3-E1 pre-osteoblastic cells were assessed. The TNrs were also implanted into the bone defects in rabbits to test their application in promoting bone formation and osteointegration at the implant-bone interface. TNrs with the medium density were found to show the best capability in promoting the protein adsorption from surrounding medium, which in turn efficiently enhanced osteogenic differentiation *in vitro* and osteointegration *in vivo*. Our work suggests that growing TNrs with a medium density on the surface of traditional Ti implants is an efficient and facile method for promoting bone formation and osteointegration in bone repair.

Ti and its alloys have been used as standard bone implants in bone repair due to their light weight^1^,^2^,^3^, chemical stability^4^,^5^,^6^,^7^ and easy processing^8^. However, as an inert non-biological material^9^,^10^,^11^, Ti cannot efficiently promote new bone formation on the implant surface, which is required for good integration with the surrounding bone tissue. Recently, it has been found that surface topography could facilitate the cell adhesion and osteogenic differentiation^12^,^13^,^14^,^15^. Inspired from this fact, we hypothesize that a Ti implant modified with the appropriate osteogenic nanostructures will promote the osteointegration by enhancing bone formation around the implant. To test such hypothesis, here we grew Ti nanorod arrays (TNrs) with different surface densities on the surface of Ti implants and evaluated whether the TNrs could promote the bone formation *in vitro* and *in vivo* for achieving osteointegration (Fig. 1).

## Results and Discussion

### Characterization of TNrs

**Figure 2:**
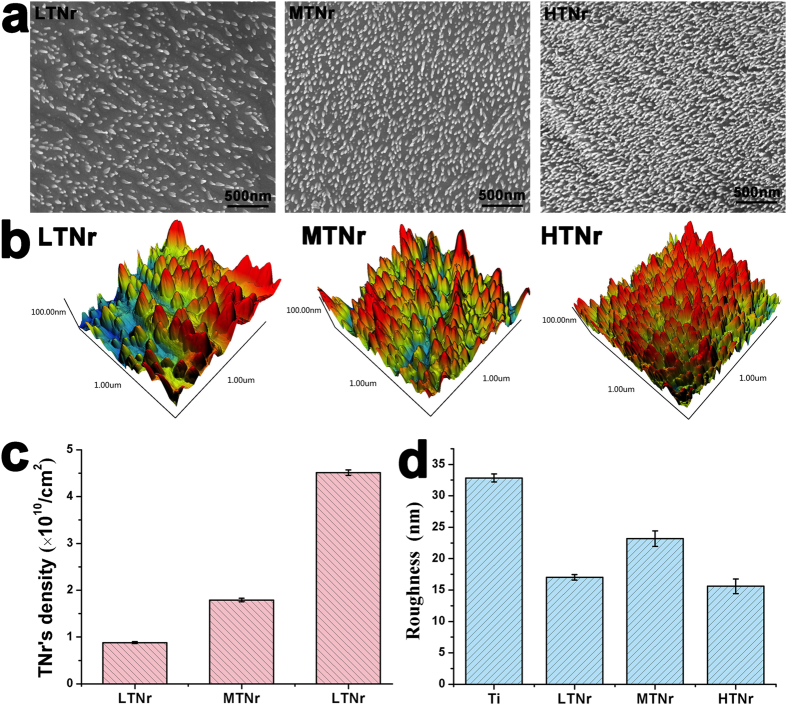
Evaluation of the TNrs in terms of density, morphology and roughness. SEM (**a**) and AFM (**b**) images of the TNrs with low density (LTNrs), medium density (MTNrs) and high density (HTNrs). (**c**) The density of TNrs. (**d**) The roughness of Ti and TNrs determined by AFM.

TNrs with tunable density and consistent chemical constitution were fabricated via anodization^16^ by controlling reaction time. Specifically, they were prepared by applying a constant current of 200 mA over the time period of 10–130 min (Fig. S1) in the electrolyte of a mixture of 1.45 wt% NH_4_F and 1.93 wt% H_2_C_2_O_4_ at room temperature. By controlling the anodization time for 15, 20 and 40 min, we prepared three samples with the average density of (0.88 ± 0.02) × 10^10^/cm^2^ (low-density TNr, LTNr), (1.79 ± 0.04) × 10^10^ /cm^2^ (medium-density TNr, MTNr), and (4.51 ± 0.06) × 10^10^ /cm^2^ (high-density TNr, HTNr), respectively (Fig. 2c). The density of TNrs was calculated by counting the number of nanorods in 500 nm^2^ of six random areas on SEM images. The length and diameter of TNrs were about 100 and 20 nm, respectively, which were calculated by averaging 20 random TNrs on SEM images using the SEM software.

The roughness of TNrs was calculated by AFM as 32.84 ± 0.64 nm (Ti, the control without nanorods), 17.02 ± 0.44 nm (LTNr), 23.18 ± 1.24 nm (MTNr) and 15.60 ± 1.17 nm (HTNr), respectively (Fig. 2d). The roughness was measured and calculated by averaging the data from 4 randomly selected areas (1 μm × 1 μm) on the substrates. The results of energy dispersive spectroscopy (EDS), electron probe micro-analysis (EPMA) and x-ray diffraction (XRD) (Fig. S2) indicated that TNrs shared the same chemical component (Ti) as the Ti sheet before nanorod growth, which was discussed in detail in our early research^17^.

### Effect of TNrs in Protein adsorption and Cell adhesion

**Figure 3:**
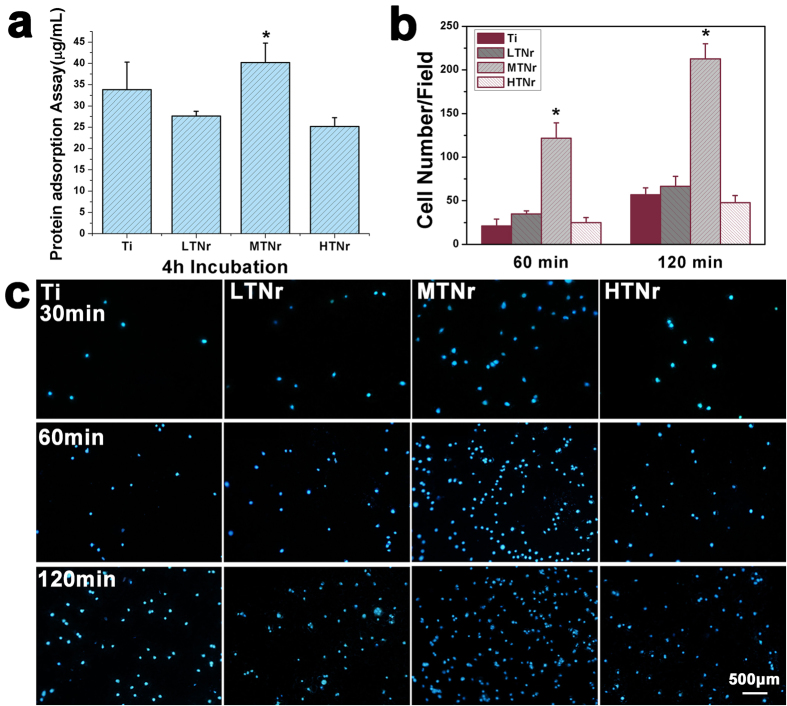
The protein adsorption and fluorescence images of MC3T3-E1 pre-osteoblasts seeded on Ti with different nanorod densities. (**a**) The amount of protein adsorption on different substrates. The specimens are incubated in α-MEM (10% FBS) for 4 h to assay the early protein adsorption. n = 4. (**b**) The cell density on different substrates. The histogram for each specimen of counted cells after 30, 60 and 120 min of incubations. n = 5. (**c**) pre-osteoblast adhesion on the different substrates measured by counting cells stained with DAPI on a fluorescence microscope after 30, 60 and 120 min of incubations. All data are reported as the mean ± standard deviation, *p < 0.05 and **p < 0.01 compared with the pure Ti, LTNr and HTNr surfaces.

TNrs along with their control (pure Ti) were incubated in α-MEM (10% FBS) for 4 h to assay the early protein adsorption behaviors on different samples (Fig. 3a and Figs S3 snd S4, Supporting Information). The protein adsorption was found to be 33.83 ± 6.45 μg/mL (Ti), 27.62 ± 1.14 μg/mL (LTNr), 40.20 ± 4.55 μg/mL (MTNr) and 25.20 ± 2.05 μg/mL (HTNr), respectively. It was obvious that the MTNr surface adsorbed statistically more protein than other surfaces (*p < 0.05). However, the adsorbed protein on the LTNrs and HTNrs was less than that on the pure Ti. When bovine serum albumin (BSA) and immunoglobulin G (IgG) were labeled with a red Cy3 dye and then interacted with different substrates followed by washing, fluorescence microscopy further confirmed that MTNrs could adsorb these two proteins with the highest efficiency (Figs. S3 and S4).

Cell adhesions on the samples after 30, 60 and 120 min of incubations were measured by counting pre-osteoblastic cells stained with DAPI. The results demonstrated that the cell number on MTNr surface was always the largest at all time intervals and statistically larger than the Ti, LTNr and HTNr surfaces (*p < 0.01) (Fig. 3b,c). However, the cell number showed no statistical difference between the Ti, LTNr and HTNr surfaces after incubation at 30, 60 and 120 min.

### Cell proliferation and differentiation

**Figure 4:**
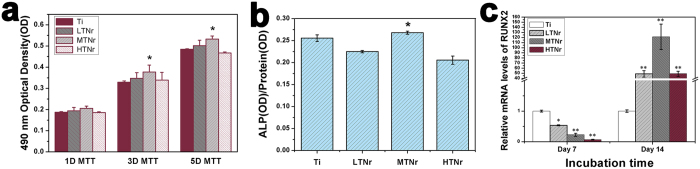
Analysis of the cell proliferation and differentiation on the surface of different substrates (Ti, LTNrs, MTNrs and HTNrs). (**a**) Proliferation (MTT assay) of pre-osteoblasts on different substrates after incubation for 1, 3 and 5 days (n = 4). (**b**) Differentiation (ALP activity) of MC3T3-E1 cells on different substrates after 14 days of culture (n = 4). (**c**) The Runx2 gene expression on TNrs by primary pre-osteoblasts after incubation of 7 and 14 days. *p < 0.05 compared to pure Ti. **p < 0.01 compared to pure Ti.

The attachment of pre-osteoblastic cells (MC3T3-E1) on the sample surfaces was analyzed for understanding the cell–materials interaction. Fig. S5 exhibited SEM images of the cells on the pure Ti and TNr surfaces after 24 h of the cell culture. It could be observed that the number of cells was larger on TNr surfaces than on the pure Ti. The shapes of the pre-osteoblasts cultured on the different surfaces were different. Cells showed more and longer filopodials stretched on TNr surface, especially on MTNrs, indicating that the medium density of TNrs was more beneficial for cell attachment. By MTT assay (Fig. 4a), MC3T3-E1 cells were found to proliferate faster on the MTNrs than on the other substrates on day 3 and 5. Collectively, pronounced cell density, proliferation and superior cell attachment were observed on MTNr substrate compared with the other substrates (Figs. 3 and 4).

Alkaline phosphatase (ALP) activity normalized to total protein content was measured to investigate the osteogenic function of the pre-osteoblasts on the pure Ti and TNrs (Fig. 4b). ALP is an early marker of osteoblast differentiation and plays a major role in mineralization^18^. Figure 4b demonstrated that MTNrs elicited an up-regulation of ALP activity compared to the rest of the substrates. On the other hand, LTNrs and HTNrs have statistically lower ALP activity than pure Ti. These data suggest that MTNrs could most efficiently promote osteogenic differentiation. This finding was further supported by the real-time PCR analysis of another osteogenic marker, Runx2 (Fig. 4c). The TNrs surface showed significantly higher gene expression (**p < 0.01) than pure Ti after incubated 14 days, and the expression on the MTNrs was the highest.

### *In vivo* test

**Figure 5:**
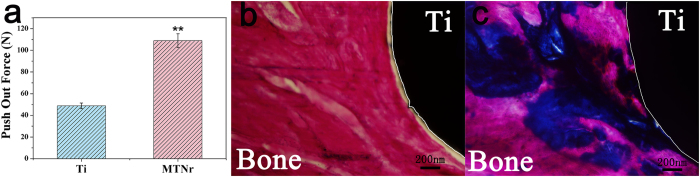
Analysis of newly formed bone on the surface of cylindrical rods modified with nanorods with a medium density. (**a**) Push out forces of Ti and MTNrs after implanted into rabbit 12 weeks. This data is reported as the mean ± standard deviation. **p < 0.01 compared with the pure Ti surface. (**b**,**c**) Masson staining of the sections of implants showing MTNrs (**c**) can efficiently induce the formation of new bone tissue in comparison with pure Ti (**b**).

The MTNrs and pure Ti controls were respectively implanted into a rabbit bone defect. In the experiment, 8 New Zealand white adult rabbits (half males and half females) aged approximately 6 months and weighing 2.3–3.0 Kg was used. Ti or MTNr (Φ3 mm × 8 mm) was implanted into the defect randomly selected around the New Zealand white rabbit leg (Fig. S6). Twelve weeks after implantation, the newly formed bone was examined by push-out test and histological analysis. The bonding strengths of different bone implants were tested by the push-out method. The results of the push-out test were expressed as the maximum push-out force in Fig. 5a, which was consistently higher for the MTNrs than for the pure Ti. The maximal push-out test force of the MTNrs was increased by 1.5-fold at 12 weeks (**p < 0.01). These results indicate that MTNrs promoted the bone formation around the implants and the consequent osteointegration.

The collagen formation on MTNrs and pure Ti were examined using Masson’s trichrome staining. Blue stained dense collagen matrix was observed on the MTNrs (Fig. 5c), while sparse collagen matrix was observed on pure Ti (Fig. 5b). It could be seen from the obvious gap that there is no new bone formation at the interface between pure Ti and bone tissue after implantation into rabbits for 12 weeks (Fig. 5b). However, collagen matrix could be observed on MTNrs after being implanted for 12 weeks (Fig. 5c). These results further confirm the good osteointegration achieved by MTNrs.

Recent *in vitro* and *in vivo* studies have confirmed the biological sensitivity to the level of nanostructured surfaces of implant biomaterials^19^. Nanotopography has been demonstrated to play an important role in modulating osteogenic tissue development^20^,^21^. In this study, Ti, LTNrs, MTNrs and HTNrs were investigated for cellular response. When a biomaterial was implanted into the body, various kinds of proteins from body fluids would be adsorbed to the substrate surface^22^. Nano-scale surface was believed to greatly contribute to the protein adsorption, which was generally considered as an important process for primary cell adhesion^23^. Up to now, the controllable creation of nanotopographic features on Ti surface is still a great challenge. Cell adhesive proteins can provide attachment sites for osteoblasts, which can lead to faster bone in-growth and implant stabilization. Our results showed that the MTNr surface adsorbed statistically more protein than other surfaces (*p < 0.05) whereas the protein adsorbed on the LTNrs and HTNrs was less than the pure Ti. Real-time PCR analysis of markers of Runx2 was further performed to confirm that the surface topography promoted osteogenic differentiation (Fig. 4c). We believe that the capability of adsorbing most proteins onto the MTNrs among all substrates studied resulted in the highest differentiation and most pronounced bone formation and osteointegration by MTNrs.

In summary, this study was to clarify the effect of the density of TNrs on MC3T3-E1 pre-osteoblastic cells behaviors and osteointegration. TNrs with the medium density were found to promote the protein adsorption, cell attachment, cell proliferation and cell differentiation. Such promotional effect of the MTNrs further promoted the bone formation and integration between the implants and the surrounding bone tissue, which was supported by push-out test and Masson staining. This work suggests that the creation of TNrs with a medium density on Ti implant surface by anodizing is a promising method of enhancing the bone formation and osteointegration.

## Methods Section

### Preparation of TNrs with tunable surface densities

Pure Ti sheets (0.1 mm thick, obtained according to standard American Society for Testing & Materials F67-2000 for biomedical application, Baoji Qichen New Material Technology Co., Ltd) with the dimension of 3.5 cm x 3.5 cm was degreased by sonicating in acetone and ethanol, then treated with 1:1 (v/v) HF and HNO_3_ solution. Treated Ti sheets were washed by deionized water and then dried with nitrogen stream.

Electrochemical anodization was conducted in a two-electrode configuration with the electrolyte of NH_4_F and H_2_C_2_O_4_ mixture, while Ti and Cu foils worked as the working and counter electrodes. Ti sheets were contacted with a Cu plate and then pressed against an O-ring, leaving a 3 cm × 3 cm surface exposed to the solution. Before electrochemical treatment, the Ti sheet was placed in the fluoride-containing solution for 10 min. Anodization was performed by applying 200 mA constant current at room temperature via a DC power supply. The as-prepared specimens were thoroughly washed with deionized water.

### Characterization of TNrs

Field emission scanning electron microscopy (FESEM, Nova Nano SEM 430, Germany) was employed to characterize the morphology of TNrs, and EDS was adopted for comparing their elements. Atomic force microscopy (AFM, SPM-9600, Shimadzu) was carried out with the scan rate and size of 0.8 Hz and 1 × 1 μm^2^, respectively, to analyze the roughness of TNr surface.

### Protein adsorption assay

A 1 mL droplet made of α-MEM (Invitrogen) and 10% Fetal Bovine Serum (FBS) (Invitrogen) was pipetted onto each specimen placed in a 24-well plate. After incubation at 37 °C for 4 h, the cells were washed three times with phosphate buffered saline (PBS) and then lysed to release proteins from samples in 0.2 vol% Triton X-100 at 4 °C for 12 h. The protein concentration in the collected solutions was determined using a MicroBCA protein assay kit (Pierce).

### Cell culture, proliferation and differentiation

Pre-osteoblasts (MC3T3-El) (Type Culture Collection of the Chinese Academy of Sciences) were cultured in α-MEM (Invitrogen) and 10% Fetal Bovine Serum (FBS, Invitrogen) media, which was replaced at regular intervals. The square samples with 10 mm in length and 0.1 mm in thickness were settled at the bottom of 24-well polystyrene culture plates with 10^4^ cells per well. All cultures were incubated at 37 °C in a humidified incubator with 5% CO_2_.

For FESEM observation, cells were fixed with 3% glutaradehyde for 6 h after co-cultured with samples for 24 h. Samples were dehydrated in a graded series of ethanol (50%, 70%, 95% and 100%) after being rinsed three times with PBS, with each concentration twice for 10 min. They were then dried and coated with gold by sputtering.

Cells were seeded on the substrates and allowed to attach for 60 and 120 min. At each prescribed time point, the non-adherent cells were removed by rinsing with PBS solution. Cells were fixed and stained with 40, 60-diamidino-2-phenylindole (DAPI). The cell numbers in five random fields were counted under a fluorescence microscope.

MTT (3-(4,5-dimethylthiazole-2-yl)-2,5-diphenyl tetrazolium bromide) assay was employed to estimate the density of viable cells^24^,^25^,^26^. After co-cultured with cells for 1, 3 and 5 days, the specimens were washed by PBS and transferred to a new 24-well polystyrene culture plate. The MTT solution was added to each well and incubated for another 4 h at 37 °C. Then dimethyl sulfoxide (DMSO) was added to dissolve any resulting formazan crystals and the optical density (OD) of each solution was measured at a wavelength of 490 nm using a spectrophotometer (Bio-tek).

MC3T3-E1 cells were seeded in 24-well polystyrene culture plates at the density of 5 × 10^3^ cells per well with the osteogenic medium containing 10 mM Na-β-glycerophophate and 10^−8^ M dexamethasone and 50 g/mL ascorbic acid. After co-cultured for 14 days, the cells were washed three times with PBS and lysed in 0.2 vol% Triton X-100 for 12 h at 4 ^°^C. Culture supernatants were combined with p-nitrophenyl phosphate (p-NPP) and then the absorbance was measured at 405 nm. The intracellular total protein content was determined using the MicroBCA protein assay kit. All results were normalized by protein content.

### Osteogenesis-related Runx2 gene expressions

MC3T3-E1 cells were seeded at the density of 2 × 10^4^ cells per well and cultured for 7 and 14 days. The total RNA was extracted using the TRIzol reagent (Invitrogen). RNA (1 μg) was reverse transcribed to complementary DNA (cDNA) using the Prime Script RT reagent kit (Takara). The real time polymerase chain reaction (Real-time PCR) was performed with SYBR Premix Ex Taq II (Takara) and primers for Runx2 (F: 5′-TCC AAC CCA CGA ATG CAC TA-3′; R: 5′-GAA GGG TCC ACT CTG GCT TTG-3′). The gene expression was calculated using the 2-ΔΔct method^25^ by Rotor-Gene Real-Time analysis software 6.0.

### Push-out test

To evaluate the bone-implant bonding strength for osteointegration, the push-out experiments to the bone-implant unit was performed on five implants of each group at each time point using a universal testing system (Shimadzu, AGS-10kNG, Japan). A force was applied to push the implant vertically out at a constant crosshead speed of 0.5 mm·min^−1^. The load-displacement curve was recorded for the measurement of the maximum pull-out force. All of the animal studies described in this work were performed according to the guidelines and regulations of National Institutes of Health (NIH) and their protocols were approved by Institutional Animal Care and Use Committee (IACUC) of General Hospital of Guangzhou Military Command of PLA.

### Histological examination

For observation of the interface between bone and implant, the tibias of rabbit were washed in PBS, fixed with 10% (wt/v) formalin overnight, dehydrated through a graded series of ethanol (70%, 80%, 90%, 95% and 100%, increasing every day) and then embedded in paraffin. From the paraffin blocks, 5 μm-thick serial sections were obtained by a diamond blade along the long axis of the implants. The sections were then mounted in epoxy resin and polished for Masson’s trichrome staining.

### Statistical analysis

The data were analyzed using SPSS 19.0 software. All the results were analyzed using an analysis of variance (ANOVA) to determine the level of significance. The data is reported as mean ± standard error of the mean. P < 0.05 and p < 0.01 were considered to be statistically significant and highly statistically significant.

## Additional Information

**How to cite this article**: Ning, C. *et al.* Ti nanorod arrays with a medium density significantly promote osteogenesis and osteointegration. *Sci. Rep.* **6**, 19047; doi: 10.1038/srep19047 (2016).

## Supplementary Material

Supplementary Information

## Figures and Tables

**Figure 1:**
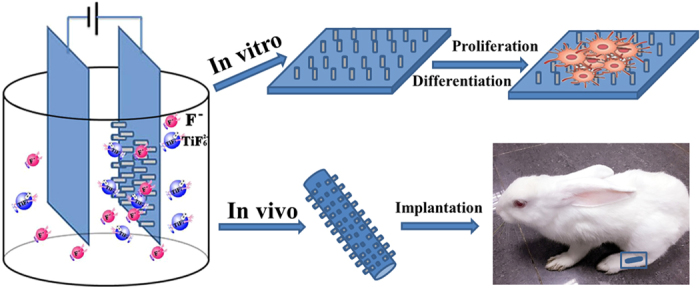
An overview of Ti nanorod arrays (TNrs) in bone repair. Left: a schematic diagram showing the fabrication of TNrs using an anodizing method. Top right: Ti and TNrs sheets are seeded with pre-osteoblasts (MC3T3-El) to study the cell response. Bottom right: Ti rods terminated with TNrs are implanted into bone defects in rabbit tibia to test their capability in promoting bone formation and osteointegration at the bone-implant interface.
