# Supplementary material for: Ti nanorod arrays with a medium density significantly promote osteogenesis and osteointegration
Source: Sci Rep. 2016 Jan 8;6:19047. doi: 10.1038/srep19047 (PMC4705471; doi:10.1038/srep19047)
Supplement: Supplementary Information [file srep19047-s1.pdf]

Supplementary Information

Ti nanorod arrays with a medium density significantly promote osteogenesis and osteointegration

Chengyun Ning, [a]* Shuangying Wang, [a] Ye Zhu,[b] Meiling Zhong, [a] Xi Lin, [c] Yu Zhang, [c] Guoxin Tan, [d] Mei Li, [c] Zhaoyi Yin, [e] Peng Yu, [a] Xiaolan Wang, [a] Ying Li, [a] Tianrui He, [a] Wei Chen, [a]` Yingjun Wang , [a]* Chuanbin Mao*[b]

[a] School of Materials Science and Engineering, South China University of Technology, Guangzhou 510641, China.

[b] Department of Chemistry & Biochemistry, Stephenson Life Sciences Research Center, University of Oklahoma,101 Stephenson Parkway, Norman, Oklahoma 73019-5300,United States

[c] General Hospital of Guangzhou Military Command of PLA, Guangzhou 510010, China

[d] Institute of Chemical Engineering and Light Industry, Guangdong University of Technology, Guangzhou 510006, China.

[e] *School of Materials Science and Technology, Kunming University of Science and Technology, Kunming 650093, China.*

[f] School of Materials Science and Engineering, Zhejiang University, Hangzhou, Zhejiang 310027, China

Corresponding authors' e-mail addresses:

Chuanbin Mao, cbmao@ou.edu

Yingjun Wang, imwangyj@163.com

Chengyun Ning, ning_lab@hotmail.com

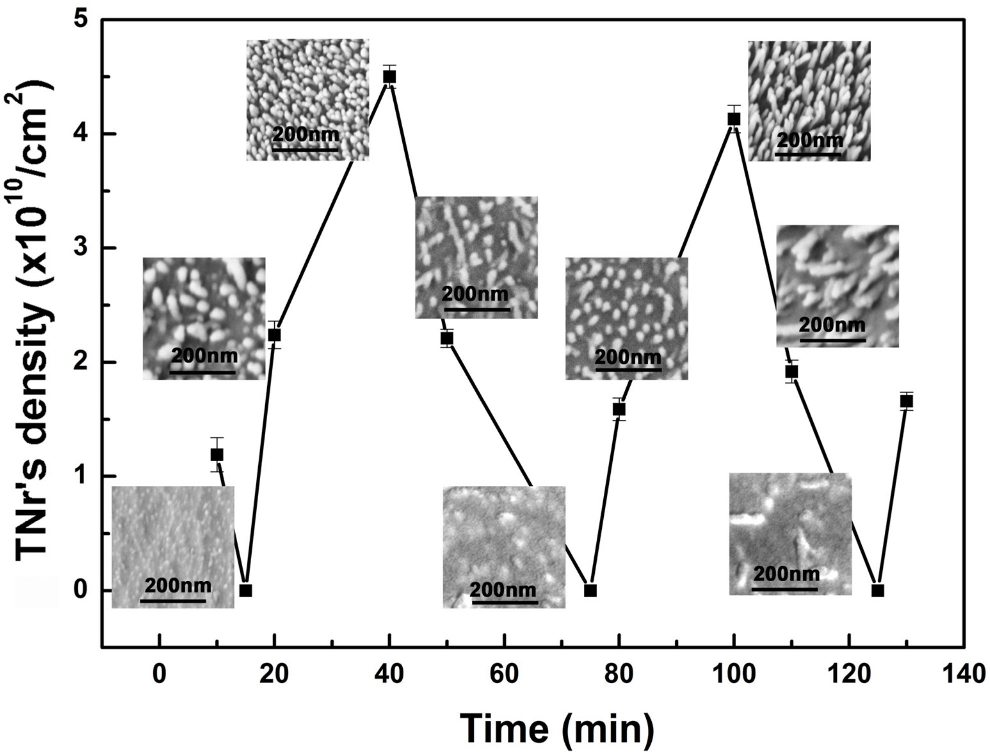

Figure S1. The density of TNrs was fabricated by anodizing over a time period of 10-130 min following a published protocol [1]. A particularly interesting feature of TNrs during galvanostatic anodization was that the TNrs density could be adjusted periodically by manipulating the anodization time. The average TNr density was considered as zero at 15 min, reached the maximum density at 40 min and the minimum density at 75 min, which constituted the first "cycle". The second cycle occurred in the range of 80-125 min with the maximum density reached at 100 min. The third cycle started at 125 min.

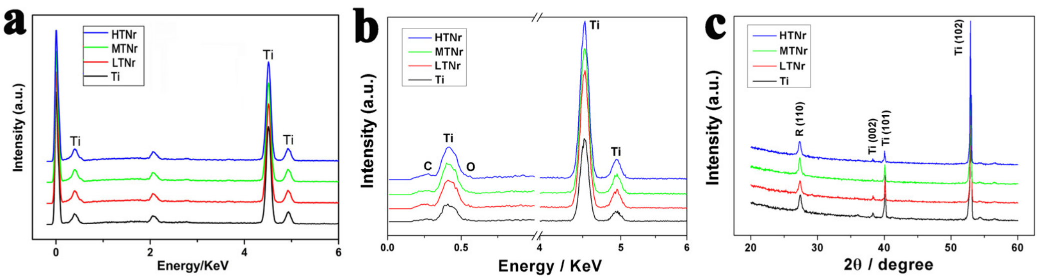

Figure S2. Evaluation of the TNrs in terms of chemical composition. EDS (a), EPMA profiles (b) and XRD patterns (c) of pure Ti, TNrs with low density (LTNrs), medium density (MTNrs) and high density (HTNrs). Note that nanorods were not scratched off the Ti foil before they were subjected to EPMA and XRD measurements. The XRD patterns of Ti and TNrs after heat-treatment at 550℃. The results showed that the TNrs shared the same chemical composition as the pure Ti.

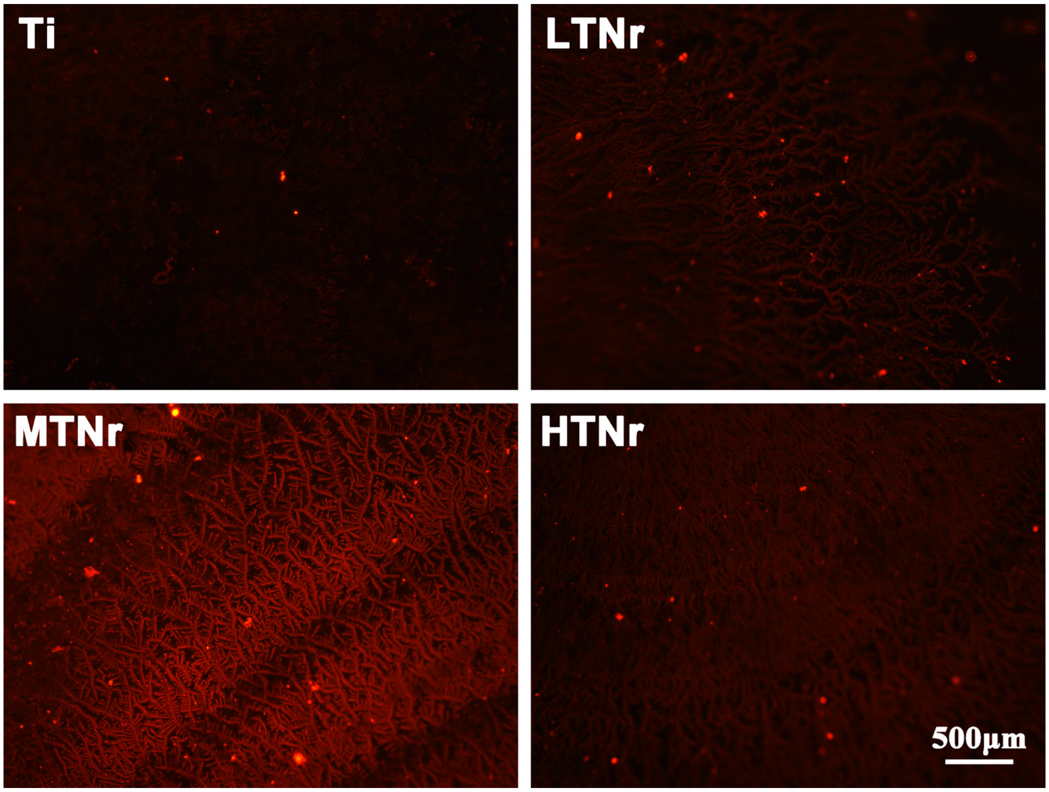

Figure S3. The fluorescent microscopy images (100 x) of BSA-Cy3 adsorption on different specimens' surfaces.

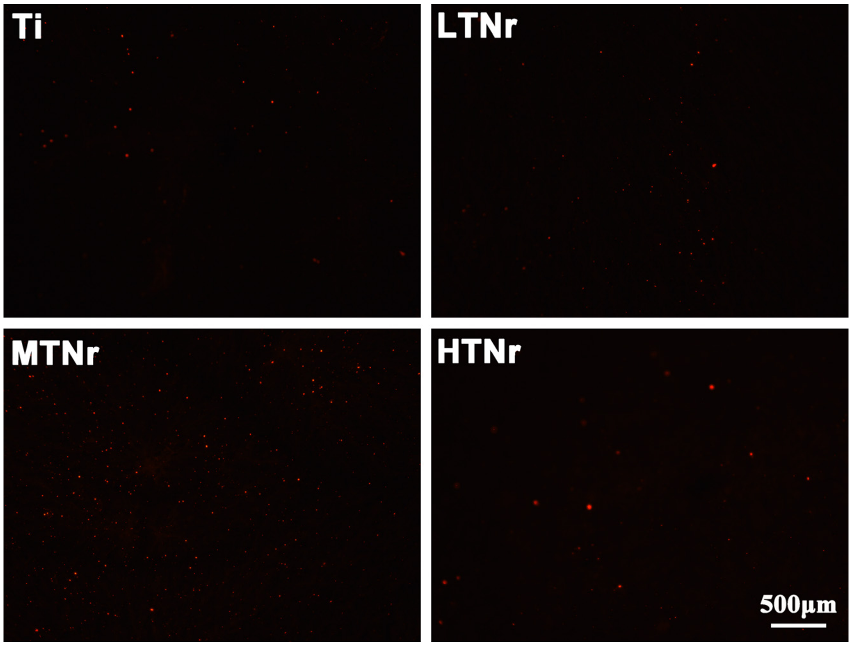

Figure S4. The fluorescent microscopy images (100 x) of IgG-Cy3 adsorption on different surfaces

The implant surfaces would adsorb a variety of proteins in vivo when implanted, especially for the nanostructured material surface. The adsorbed protein could offer focal adhesion points for osteoblast, thus promoting bone formation and resulting in better stability of the implant. This was usually regarded as an important process of early cell adhesion [2]. In addition, surface nanotopography can provide protein adsorption and cell adhesion with adhesion points, playing an important role in cell proliferation and differentiation [3]. Figure S3 and S4 were the fluorescent microscope images for pure titanium and TNrs with adsorbed BSA and IgG after 4 h, respectively. They qualitatively showed that the medium density of TNrs adsorbed the most protein. This result was consistent with the early cell adhesion.

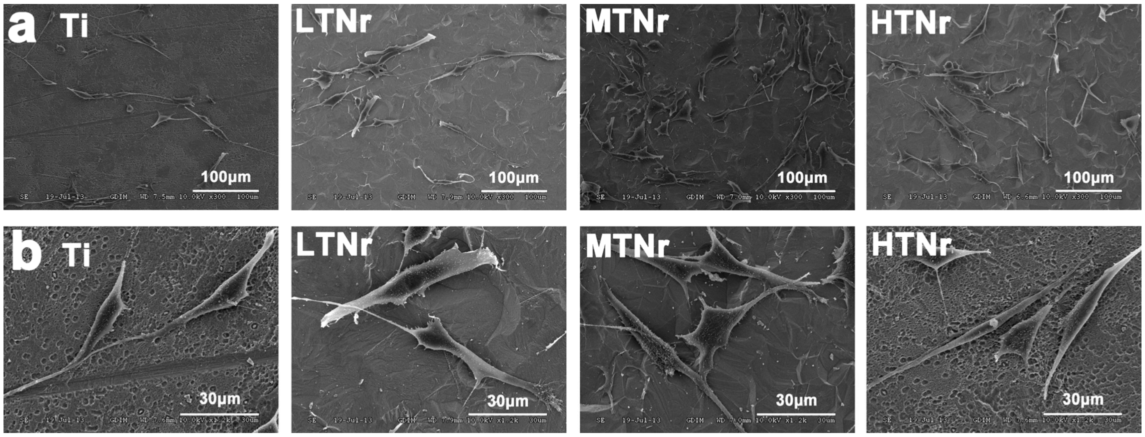

Figure S5. SEM images (a, b) of TNrs with osteoblasts showing the interaction of osteoblasts on different surfaces after 24 h of culture. b is the higher magnification of selected areas in a.

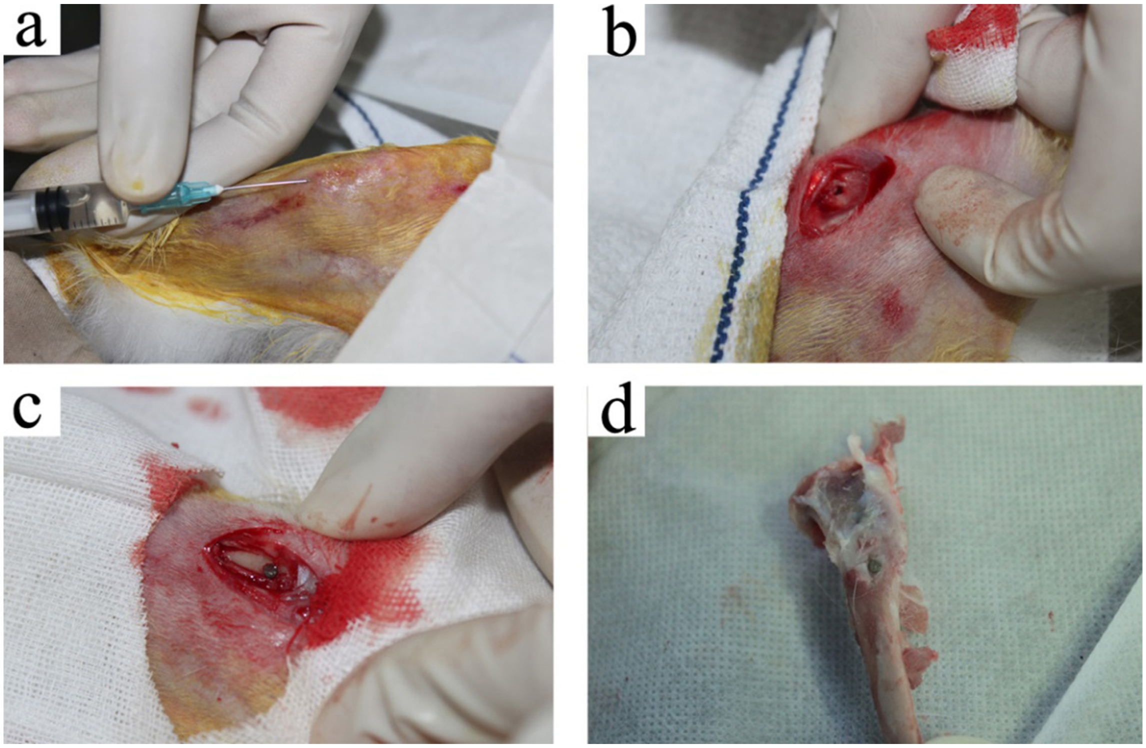

Figure S6. Animal experiment process: a: Anesthesia was given for operation; b: Tibial metaphysis was exposed and a hole was made for implants; c: Materials were implanted; d: Bone after implantation after 4 weeks.

Briefly, first, through ear marginal vein 3% pentobarbital (50 mg/kg; Guangzhou sile experimental instrument chemical co., LTD.) was injected into narcotize rabbit. The rabbit was then fixed by supine position, and then 2% lidocaine (Shanghai zhaohui pharmaceutical co., LTD) was injected into rabbit proximal tibia to achieve local anesthesia (S6a). The operation area was shaved and disinfected by iodine. The whole process (from the skin, fascia, muscles, to periosteum, step by step into the inside of the proximal tibia) was performed in accordance to the aseptic technical operation strictly. A 3 mm diameter hole was drilled in implant position (S6b), and cooled by saline flushing during drilling. A cylinder-like Ti (3 mm × 8 mm) terminated with MTNrs was implanted into the defect (S6c). The implant only penetrated the unilateral cortex. After the whole process, the incision was sutured step by step. After implantation, the rabbits were fed penicillin 40000 u every day for three days. The rabbits were put to death via ear vein air injection 12 weeks after implantation and the bones could be excised for analysis (S6d).

Reference:

1. Zhong, M. *et al*. Ti nanorod arrays with periodic density fabricated via anodic technology. *Micro Nano Lett.* **9**, 168-170 (2014).
2. Xu, L. *et al*. In vitro and in vivo evaluation of the surface bioactivity of a calcium phosphate coated magnesium alloy. *Biomaterials* **30**, 1512-1523 (2009).
3. Puleo, D. & Nanci, A. Understanding and controlling the bone–implant interface. *Biomaterials* **20**, 2311-2321 (1999).
